# Supplementary material for: Mixed Effects Models for Resampled Network Statistics Improves Statistical Power to Find Differences in Multi-Subject Functional Connectivity
Source: Front Neurosci. 2016 Apr 12;10:108. doi: 10.3389/fnins.2016.00108 (PMC4828454; doi:10.3389/fnins.2016.00108)
Supplement: Supplementary file 1 [file Presentation1.PDF]

Supplementary Material: Supplementary Material for Mixed Effects Models for Resampled Network Statistics Improve Statistical Power to Find Differences in Multi-Subject Functional Connectivity

Manjari Narayan [1,*] **and Genevera I. Allen** [1,2,3]

[1] *Department of Electrical and Computer Engineering,*
[2] *Department of Statistics, Rice University, Houston, TX, USA*
[3] *Jan and Dan Duncan Neurological Research Institute, Houston, TX, USA*

Correspondence*:
Manjari Narayan
Department of Electrical and Computer Engineering, Rice University,
6100 Main St, Houston , TX, 77005, USA, manjari.narayan@gmail.com

Recent Advances and Challenges on Big Data Analysis in Neuroimaging

A SUPPLEMENTARY SIMULATIONS & FIGURES

In this appendix, we provide supplementary simulations and figures to complement the power analyses and summary of type-I error control that appear in Figures 3,4 & 5 of our manuscript. The setup for the supplementary simulations follows the procedures outlined in Section 4.1. Figures A.1 & A.2 provide a complete set of type-I error simulations for node and subnetwork density, respectively, and complement the power analyses found in Figures 3 & 4. Additionally, we demonstrate the impact of sparsity on the ability of R^3, R^2 and the standard method to detect covariate effects in Figure A.3. Here, we employ the node density metric in the medium SNR case ($\nu^2 = .25$) as a representative example, while holding all other parameters consistent with Figure 3 of the manuscript constant with exception of baseline sparsity threshold τ. While the simulations in our manuscript employed realistic networks (illustrated in Figure A.0) obtained by setting all partial correlations whose absolute values were less than $\tau = .25$ to zero, we varied this threshold to values $\{.1, .4\}$ to obtain both denser and sparser baseline networks.

The supplementary simulations in Figures A.1 & A.2 are consistent with Figure 5 of our manuscript, and demonstrate that all methods approximately control type-I error at the 5% level. In Figure A.3, as expected, statistical power decreases with smaller sample sizes, especially when $t \approx p$. In the sparser baseline case, our methods, R^3 and R^2, are able to achieve better statistical power to detect covariate effects over standard F-tests. In the sparser network case, it is easier to estimate subject networks even in low sample sizes of $t \approx p$, and initial stability scores continue to discriminate between true and false edges more effectively than in denser network regimes. Since the benefits of adaptive estimation depend on initial network estimates, we observe that the random adaptive penalization component of R^3 improves the estimates of network metrics, thus achieving greater statistical power than R^2 in sparser network regimes with small sample sizes. However, when baseline networks become denser, particularly when $\tau = .10$, the ability of all methods to detect covariate effects begin to fail as within subject sample sizes reduce to $t \approx p$. Overall our supplementary simulations continue to highlight the importance of within subject sample size t, and the benefits of our methods, R^3 and R^2 over the standard approach at smaller sample sizes.

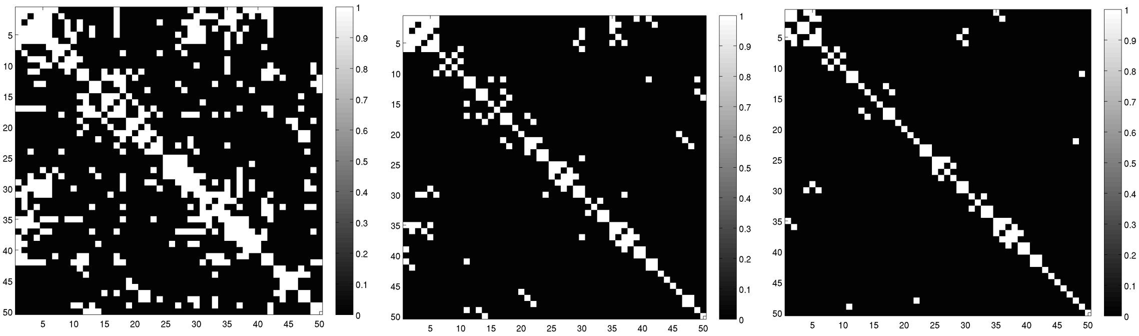

(a) Baseline Network, $\tau = .10$ (b) Baseline Network, $\tau = .25$ (c) Baseline Network, $\tau = .4$

Figure A.0 ***Empirically Derived Pseudo Real Networks.*** The simulations in the main paper in Figures 2 & 3 all begin with the baseline network (moderate density case) with threshold $\tau = .25$ in (b). Then each individual subject network is simulated as described in Section 4.1. The simulations in Figures A.3 employ sparser and denser baseline networks given by thresholds $\tau = .1$ and $\tau = .4$ for the same experiment for node density in Figure 2.

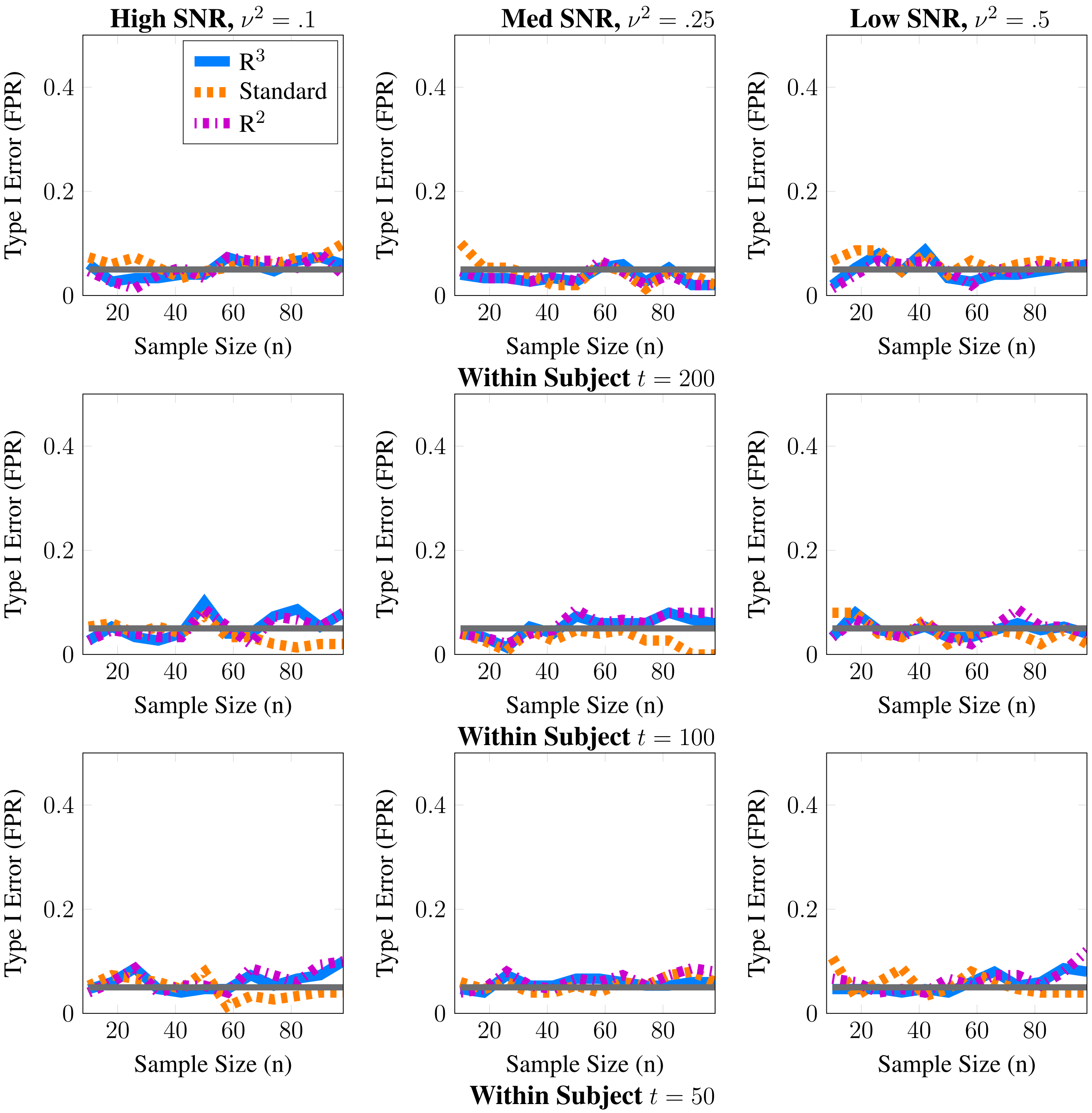

Figure A.1. ***Statistical Type I Error Control for Node Density.*** These simulations evaluate the level of our tests; we report the estimated type-I error as a function of subject sample size n. The grey line represents the 5% level of the test. Here, we provide a complete set of Type-1 error simulations to complement the power analysis in Figure 3. All methods approximately control type I error across all scenarios studied for node density.

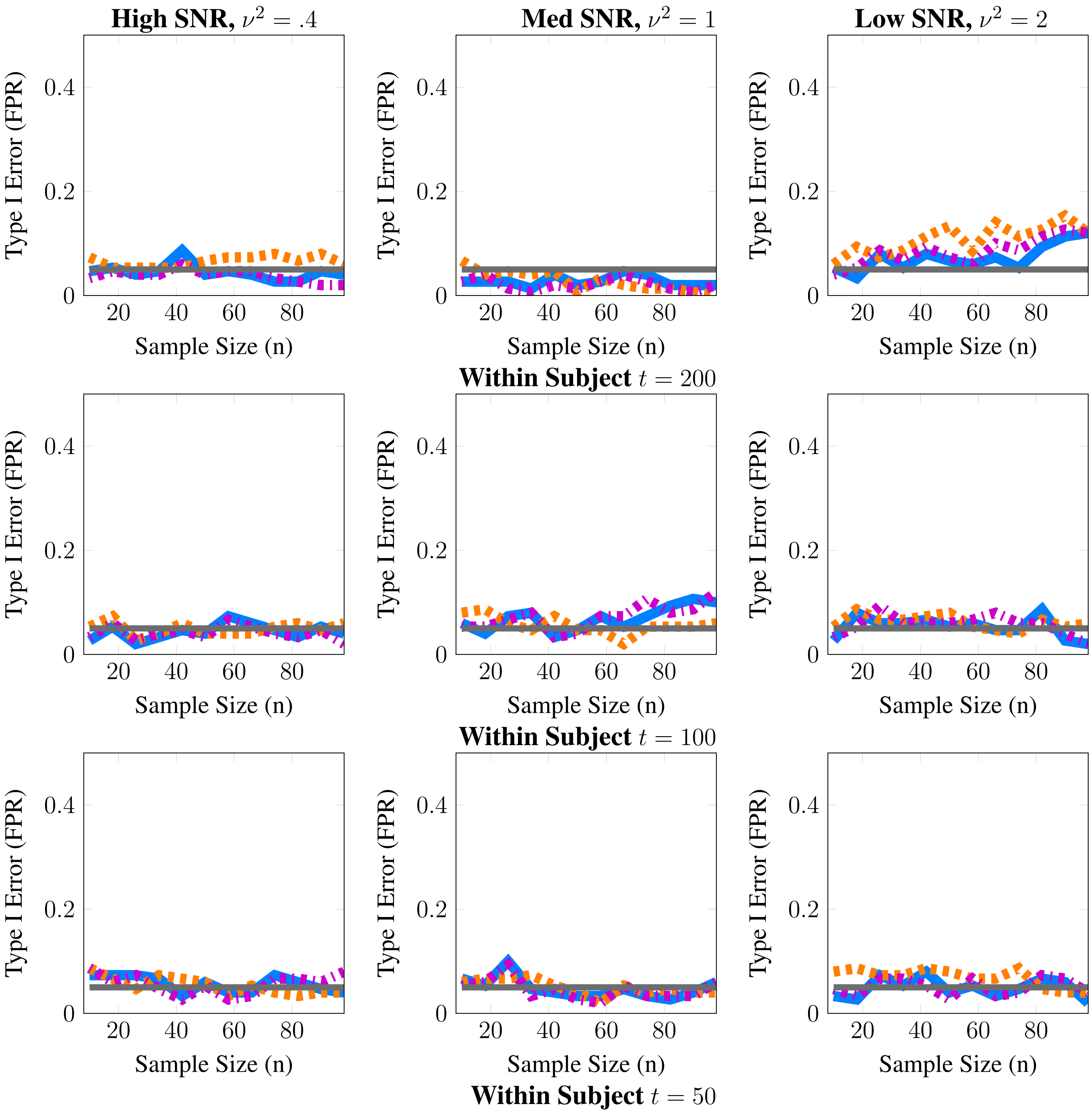

Figure A.2. ***Statistical Type I Error Control for Subnetwork Density.*** These simulations evaluate the level of our tests; we report the estimated type-I error as a function of subject sample size n. The grey line represents the 5% level of the test. Here, we provide a complete set of Type-1 error simulations to complement the power analysis in Figure 4. All methods approximately control type I error across all scenarios studied for subnetwork density.

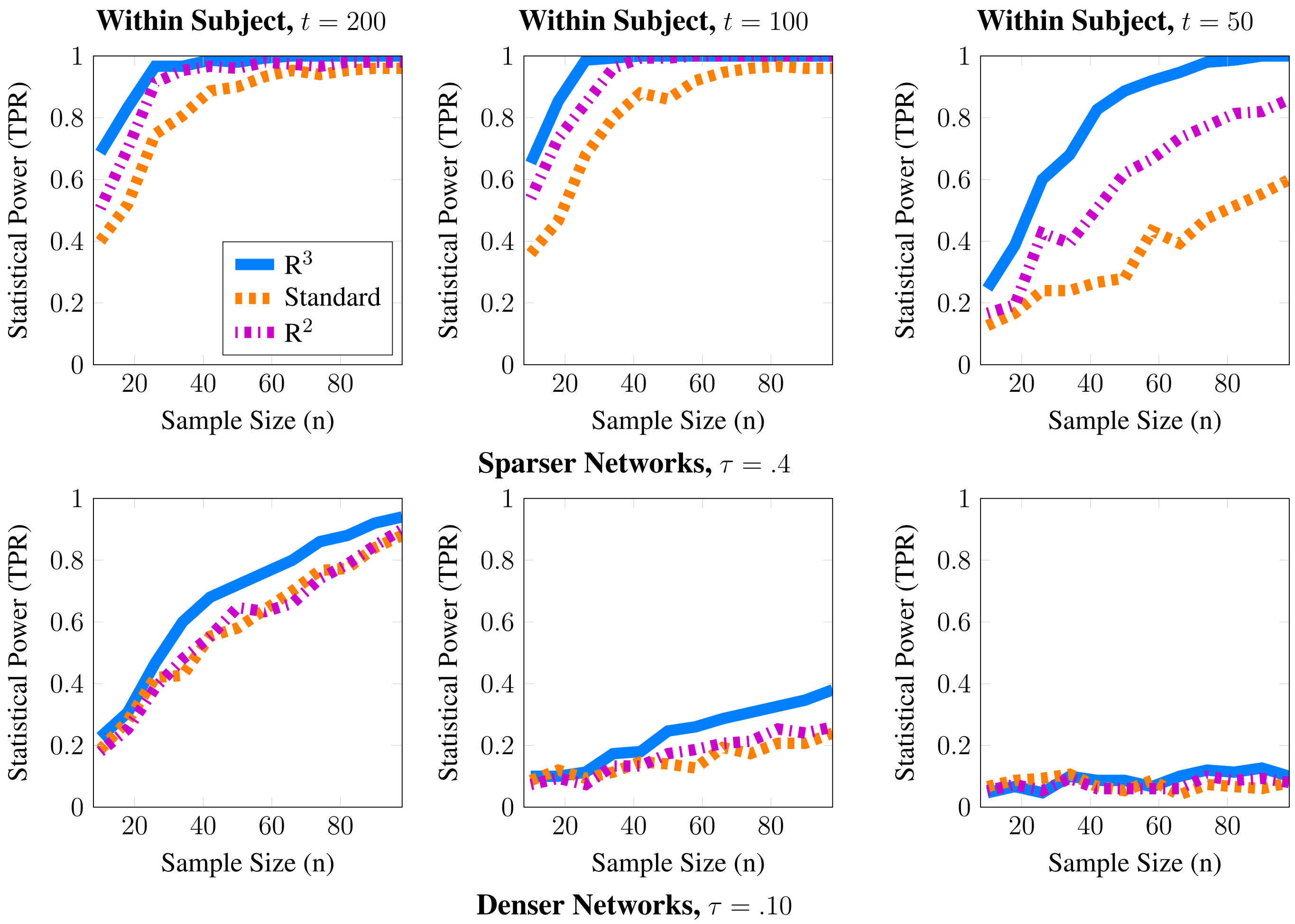

Figure A.3. ***Statistical Power Analysis with Varying Baseline Sparsity.*** This figure complements the power analysis for node density in Figure 3 of the manuscript for the medium SNR case ($\nu^2 = .25$), where the number of nodes is $p = 50$. Whereas the baseline pseudo-real networks in Figure 3 consist of edges whose absolute partial correlation strength was greater than $\tau = .25$, here we consider simulations where the baseline density is decreased ($\tau = .40$) as well as increased ($\tau = .10$). Notice that for the sparse baseline network, our results broadly match those of Figure 3. When node density varies with an explanatory covariate ($q = 1$), statistical power to detect this covariate effect improves with subject sample size n but crucially depends on the number of independent fMRI samples t from a single session. When networks are hard to estimate at limited within subject sample sizes $t \approx p$, we expect estimates of node density to be both highly variable and potentially biased. However, as long as the baseline networks are sufficiently sparse, we can account for these errors via our methods R^3 and R^2. In fact, R^3 achieves near perfect statistical power by adaptively improving the network metrics estimates of R^2, thus improving statistical power over R^2 and standard F-tests. In contrast, when baseline graphs are dense, and the sample sizes approach ($t \approx p$), it becomes impossible to detect covariate effects. Thus, within subject sample sizes continue to be crucial for detecting covariate effects

B TEST STATISTICS FOR R^3 AND R^2

R^3 and R^2 model resampled network metrics using repeated measures mixed effects models to account for two levels of variation in continuous network metrics. In this appendix, we begin with some elementary estimators and test statistics for covariate effects in the linear mixed effect (LME) model defined in Eq. (9) & (10) in Section 2.3 of our manuscript. Additionally, in Section B.2 we provide alternatives to the LME models in Section 2.3.3 of our manuscript for two levels of binary valued resampled statistics. As in the case of LME models, we outline relevant correlated binomial models and corresponding estimators for covariate effects.

B.1 ESTIMATORS FOR REPEATED MEASURES LME

Many estimators **Agresti** (2015) are available to estimate fixed effects for LME models , where the number of resamples within each subject is complete and balanced. We employ a generalization of ordinary least squares regression for correlated two-level data given by weighted least squares estimators (GLS) **Agresti** (2015). Ideally, in order to make the least square residuals independent we weight the residuals by the precision matrix, V_i^{-1}, to obtain efficient estimates of $\boldsymbol{\beta}$.

We redefine the earlier notation in Section 2.1 for the population model to account for the availability of resampled network metrics. We denote the overall design matrix by $\mathbf{W} = [W_1 \ \ldots \ W_n]^\top$. Here W_i is the $B \times (1 + q + r)$ subject level design matrix for the fixed effects, obtained by stacking centered and scaled explanatory and nuisance covariates $[X_i \ Z_i]$. Let c denote a contrast vector to separate explanatory and nuisance covariates of interest such that $c = [0 \ \mathbf{1}_{1\times q} \ \mathbf{0}_{1\times r}]$ and $c^\top[\boldsymbol{\beta} \ \boldsymbol{\gamma}] = \boldsymbol{\beta}_{\backslash 0}$. We omit the subscript excluding the intercept when referring to $\boldsymbol{\beta}_{\backslash 0}$ in this section. Here B denotes the number of resamples, n the number of subjects, q and r the number of explanatory and nuisance covariates, respectively.

Thus, the fixed effects estimate takes the form $\hat{\boldsymbol{\beta}}_{GLS} = \left(\sum_{i=1}^n W_i^\top V_i^{-1} W_i\right)^{-1} \left(\sum_{i=1}^n X_i^\top V_i^{-1} \mathbf{U}_i^*\right)$. The corresponding partial Wald statistic for explanatory fixed effects is given by

$$\mathcal{T} = \frac{\hat{\boldsymbol{\beta}}_{GLS}^\top \{\text{Var}(\hat{\boldsymbol{\beta}})_{GLS}\} \hat{\boldsymbol{\beta}}_{GLS}}{rank(c)}, \quad \text{Var}(\hat{\boldsymbol{\beta}})_{GLS} = c^\top \left(\sum_i^n (W_i^\top V_i^{-1} W_i)^{-1}\right) c \qquad \text{(B.1)}$$

Since our two level model in Section 2.3.3 is a random intercept model for repeated measures, V_i^{-1} has compound symmetry structure and depends on two unknown parameters (ν^2, ϕ^2) that do not vary with subjects i. Consequently standard ANOVA and restricted maximum likelihood estimators for variance components, ϕ, ν coincide (**Searle et al.**, 2009), given by $\hat{\phi}^{\star 2} = \frac{\sum_i \sum_b (\tilde{u}^{\star(i,b)} - \bar{\tilde{u}}^{\star(i,.)})^2}{n(B-1)}$ and $\hat{\nu}^2 = \frac{\sum_i^n (\bar{\tilde{u}}^{\star(i,.)} - \bar{\bar{\tilde{u}}}^{\star(.,.)})^2}{n}$. While Wald-type test statistics are asymptomtically χ^2 distributed, they are better approximated by scaled F-distributions at finite samples. Finite sample corrections and estimates of the degrees of freedom for these F-distributions, provided by **Kenward and Roger** (1997), are widely adopted for inference in LME models to ensure better type-I error control. For more details on computational procedures and extensions to these models for more complex experimental designs, we refer the reader to **Agresti** (2015).

B.2 MIXED EFFECTS MODELS FOR CORRELATED BINARY DATA

As in the case of continuous metrics, when R^2 and R^3 produce resampled binary network statistics per subject, our data possesses two levels of variability. Although such statistics can be summarized using proportions $\sum_{b=1}^B \tilde{u}^{*,(i,b)}$ per subject, we cannot model these correlated proportions using binomial distributions, as the binomial assumes all $nB \times 1$ binary valued resampled statistics to be independent. In fact,

we expect the resampled statistics within each subject to be positively correlated. To resolve this problem, following the well established literature (**Liang and Hanfelt**, 1994; **Agresti**, 2015), we consider two-level models for correlated binary data.

To understand binomial models for correlated data, consider the example of the probability of observing an edge as the network metric of interest. Recall, from Eq.(6) that we seek to conduct inference over the fixed effect $\boldsymbol{\beta}$ which describes the rate of change in the subject edge probability in a population logit $\pi_i = \eta_i = X\boldsymbol{\beta} + Z\boldsymbol{\gamma}$ for a unit change in the covariate (**Williams**, 1982). However we only observe network metrics for a sample of subjects in the population. To account for this inter-subject sampling variability, we introduce a continuous latent random variable P_i that takes values in the interval $[0, 1]$. Additionally, however, we do not observe individual subject edge probabilities P_i but rather observe binary network statistics per subject. Thus, conditional on a subject's true edge probability P_i, we assume that each resampled network statistic $\tilde{u}^{*,(i,b)}$ is Bernoulli distributed, such that $\tilde{u}^{*,(i,b)}|P_i = p_i \sim \text{Ber}(1, p_i)$. Together, this gives us the following model for the observed proportions $\mathbf{U}_i^* = \sum_b \tilde{u}^{*,(i,b)}$

$$
\begin{aligned}
P_i \sim \mathcal{F}, \quad E(P_i) = \pi_i,\ \text{Var}(P_i) = \phi\pi_i(1-\pi_i) \\
E(\mathbf{U}_i^*) = B\pi_i,\ \text{Var}(\mathbf{U}_i^*) = B\pi_i(1-\pi_i)[1+\phi(B-1)]
\end{aligned}
\tag{B.2}
$$

By employing this two-level model, we account for overdispersion in correlated resampled statistics in the form of the multiplicative correction term $[1+\phi(B-1)]$. Note that, while we can specify a fully parametric model for $\mathcal{F}$ using beta or correlated binomial distributions, specifying the first and second moments is adequate (**Williams**, 1982; **Searle et al.**, 2009) for the estimation and inference of fixed effects.

In the presence of balanced within subject resamples B, our two-level model (B.2) is very similar to our single level logistic-linear model in (**??**) with the exception of the additional overdispersion factor $(1+\phi(B-1))$. Thus, standard iterative reweighted least squares estimation can be used to obtain estimates of fixed effects $\boldsymbol{\beta}, \boldsymbol{\gamma}$ and moment estimators for ϕ (**Kleinman**, 1973; **Williams**, 1982). We proceed with inference for $\hat{\boldsymbol{\beta}}$, using Wald type statistics in (B.1), by ensuring that standard sample variance estimates for $\text{Var}(\hat{\boldsymbol{\beta}})$ incorporate the overdispersion factor. In the absence of balanced data, or for more complex experimental designs such as longitudinal imaging studies we recommend the maximum quasi-likelihood or generalized estimating equations (**Liang and Hanfelt**, 1994) for correlated binary data.

REFERENCES

Agresti, A. (2015), Foundations of Linear and Generalized Linear Models (John Wiley & Sons)

Kenward, M. G. and Roger, J. H. (1997), Small sample inference for fixed effects from restricted maximum likelihood, *Biometrics*, 983–997

Kleinman, J. C. (1973), Proportions with extraneous variance: single and independent samples, *JASA*, 68, 341, 46–54

Liang, K.-Y. and Hanfelt, J. (1994), On the use of the quasi-likelihood method in teratological experiments., *Biometrics*, 50, 3, 872–880

Searle, S. R., Casella, G., and McCulloch, C. E. (2009), Variance components, volume 391 (Wiley-Interscience)

Williams, D. A. (1982), Extra-binomial variation in logistic linear models, *Applied statistics*, 144–148
